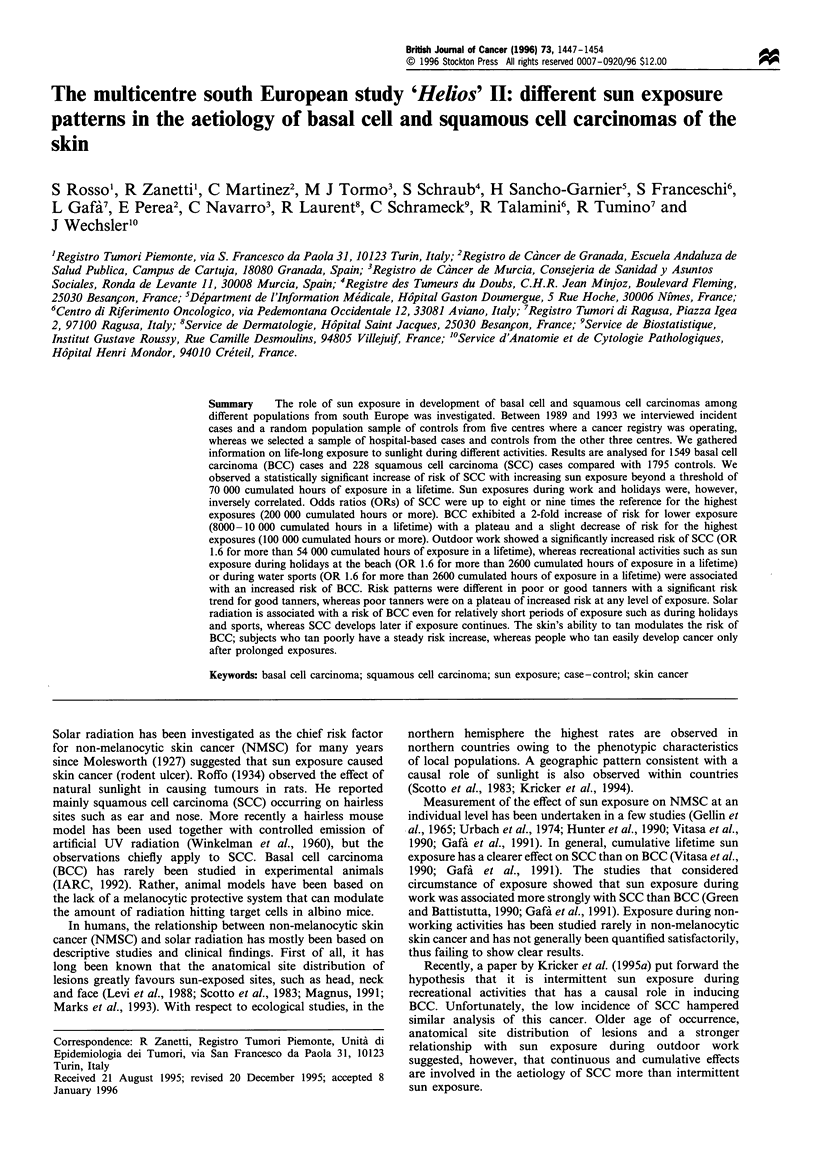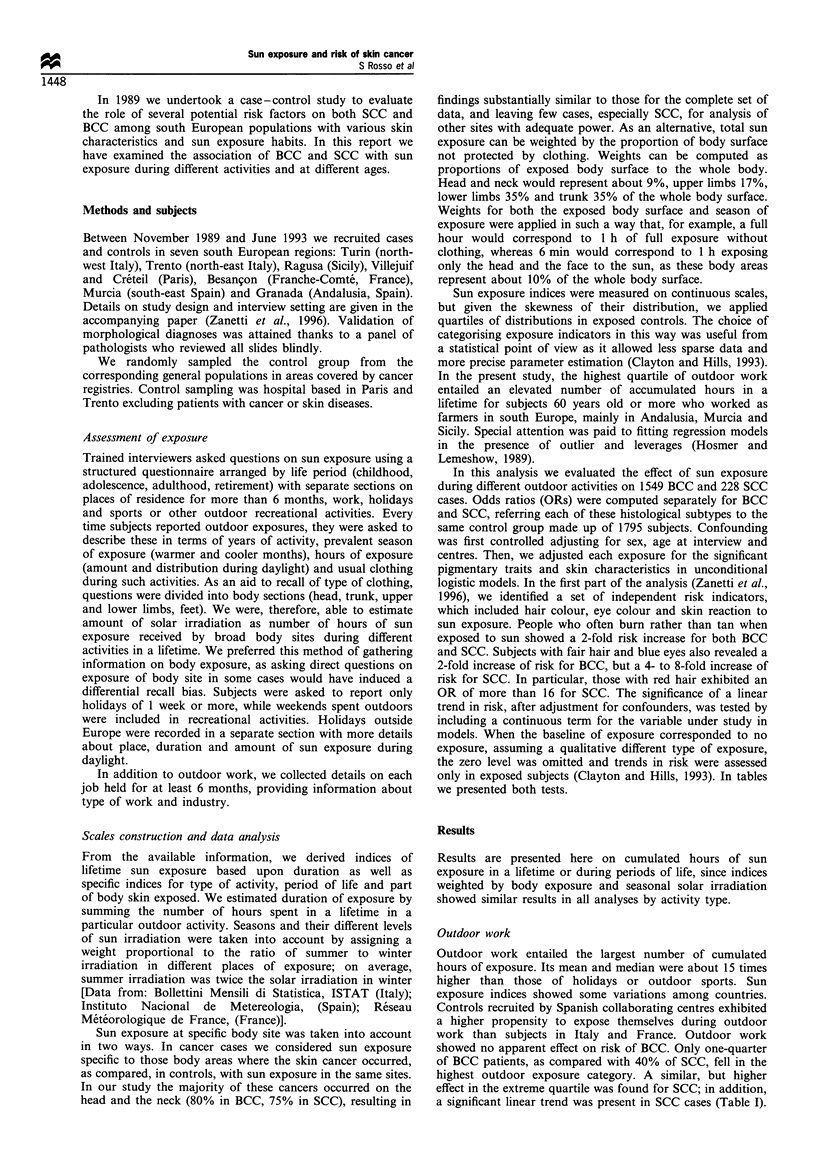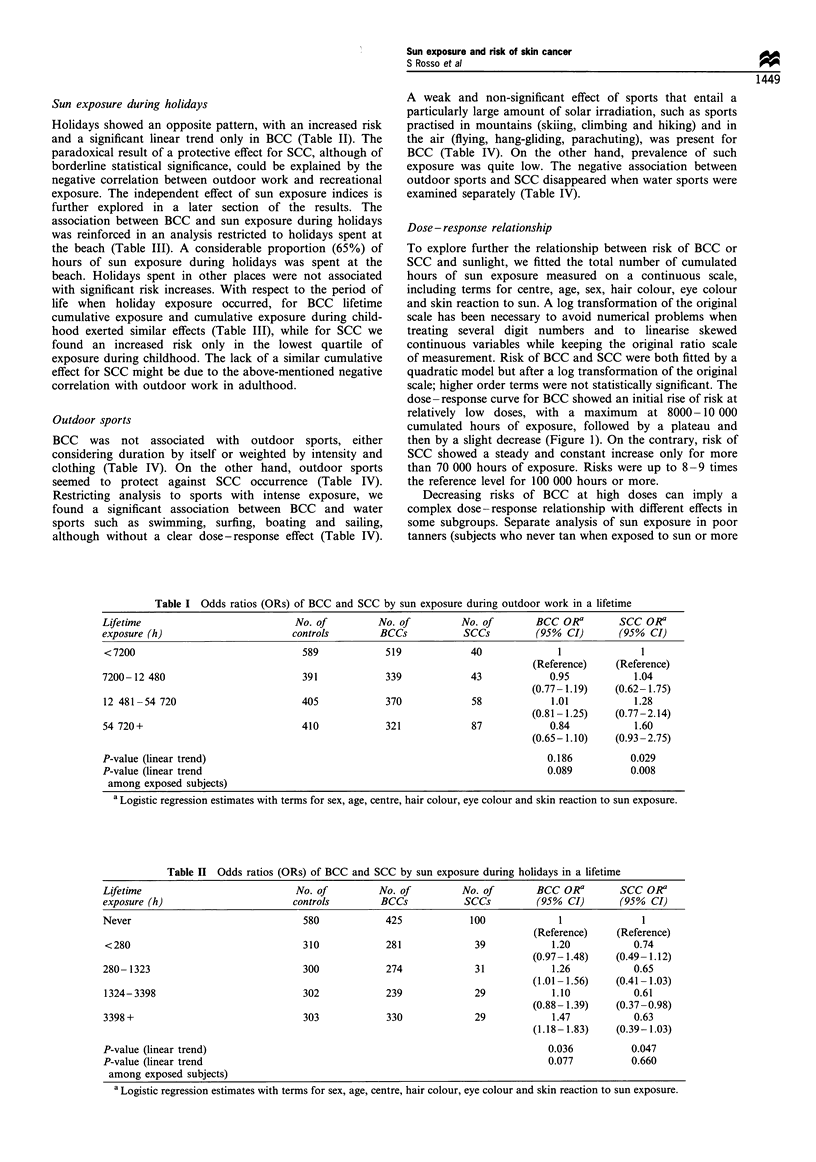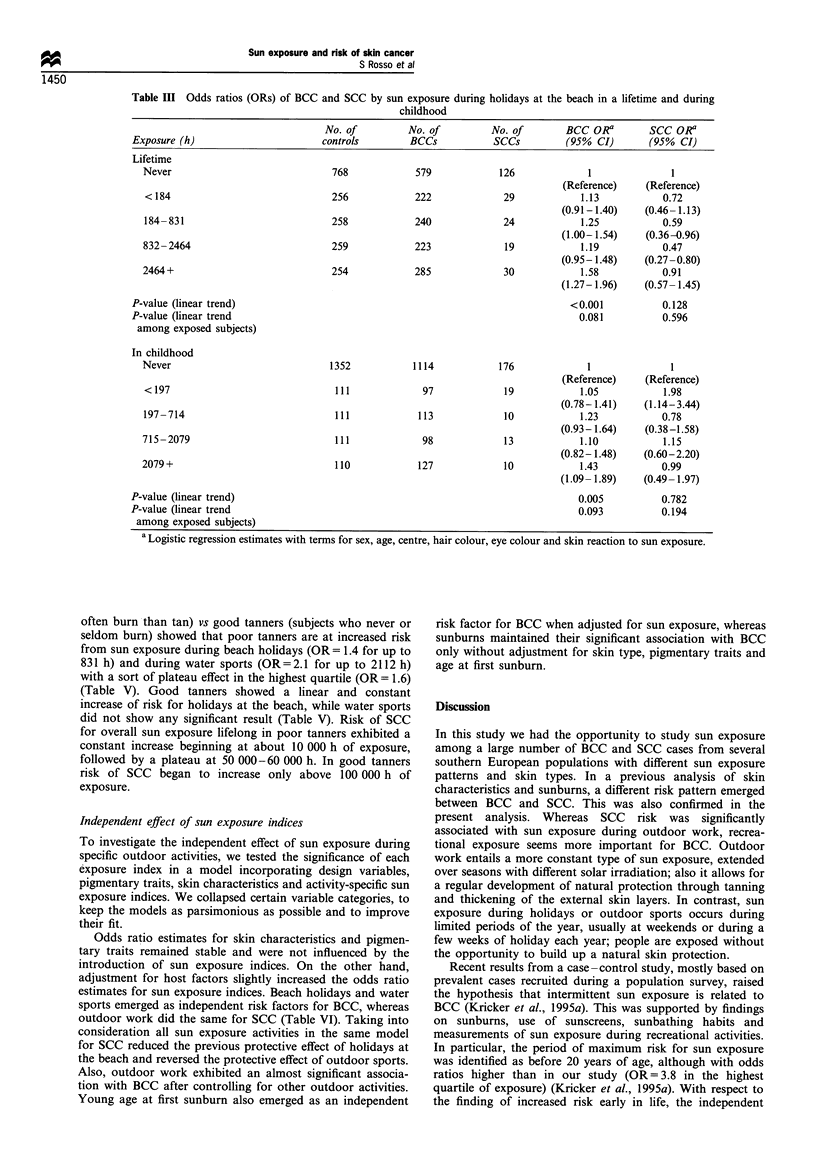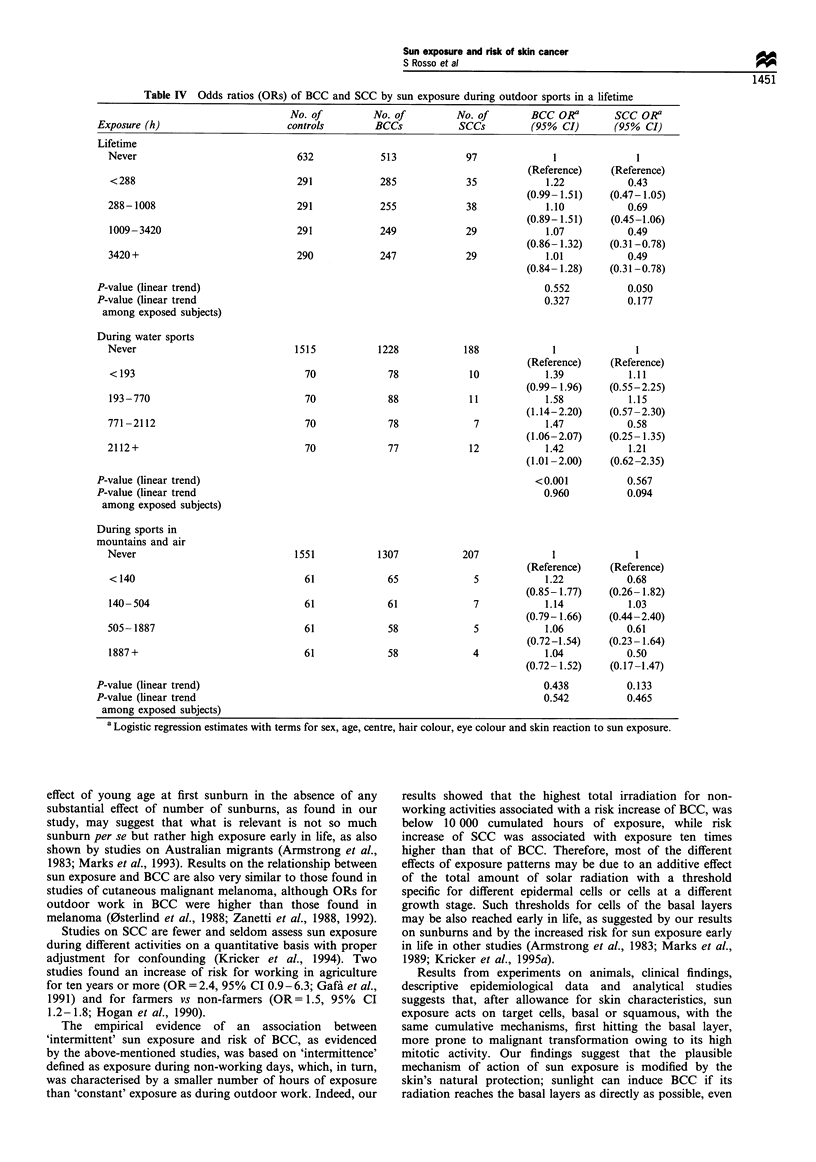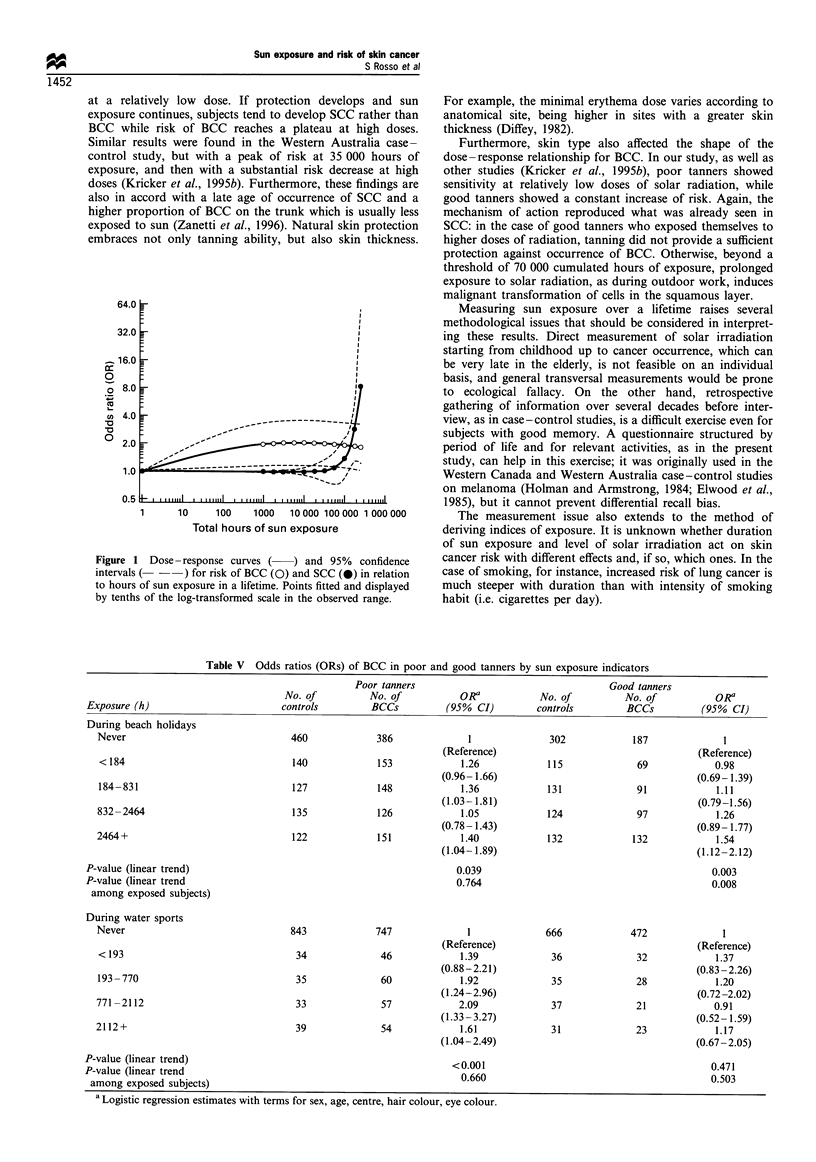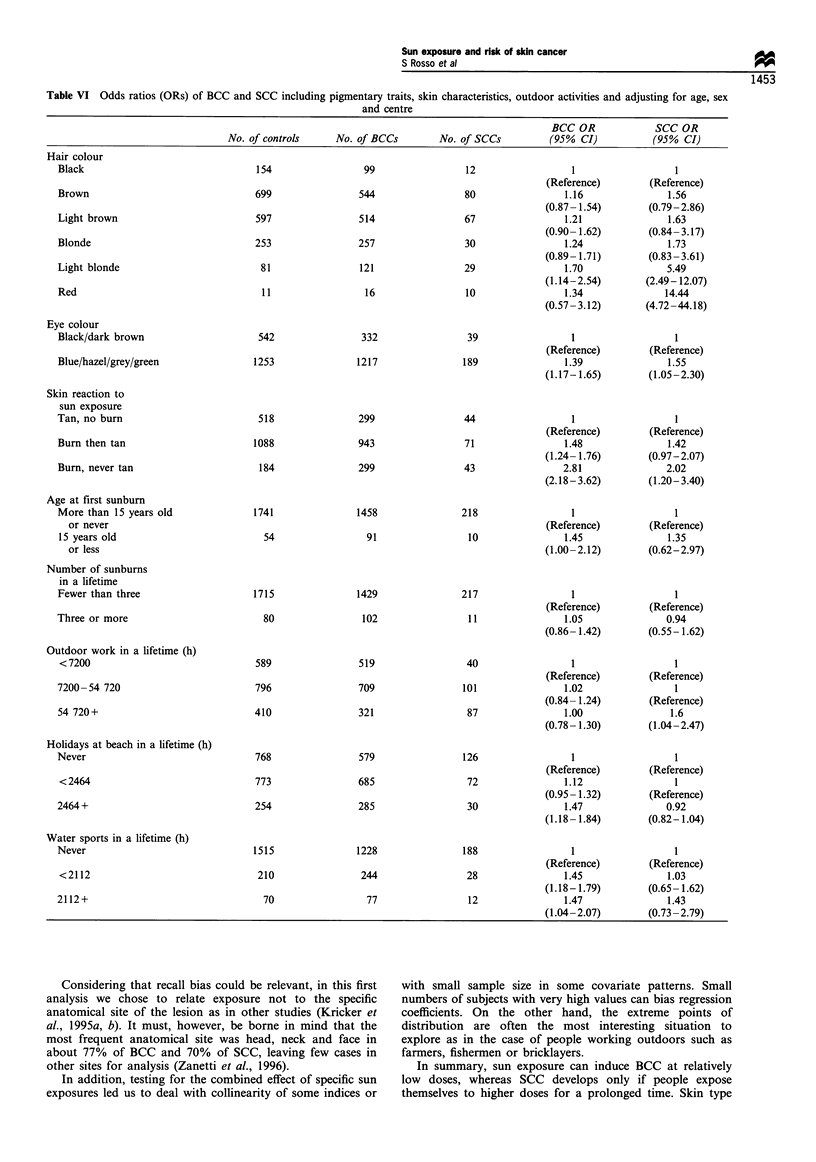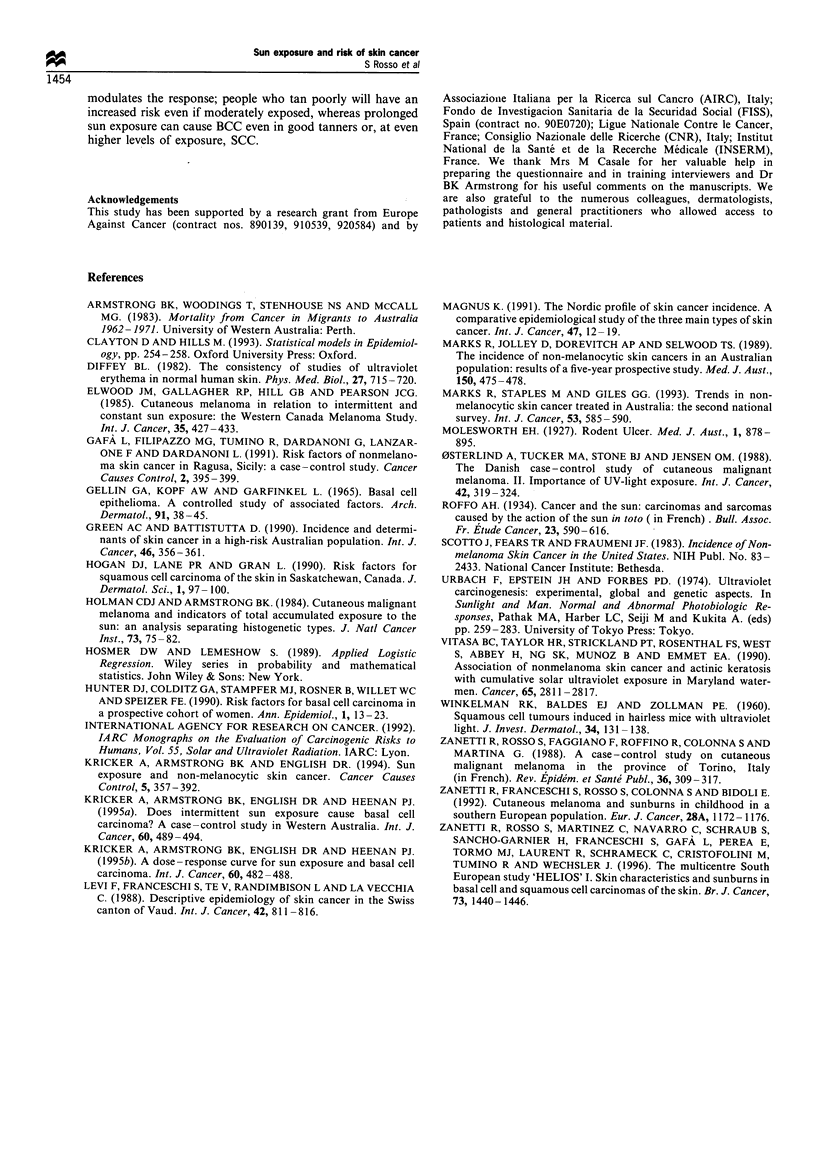# The multicentre south European study 'Helios'. II: Different sun exposure patterns in the aetiology of basal cell and squamous cell carcinomas of the skin.

**DOI:** 10.1038/bjc.1996.275

**Published:** 1996-06

**Authors:** S. Rosso, R. Zanetti, C. Martinez, M. J. Tormo, S. Schraub, H. Sancho-Garnier, S. Franceschi, L. Gafà, E. Perea, C. Navarro, R. Laurent, C. Schrameck, R. Talamini, R. Tumino, J. Wechsler

**Affiliations:** Registro de Càncer de Granada, Escuela Andaluza de Salud Publica, Spain.

## Abstract

The role of sun exposure in development of basal cell and squamous cell carcinomas among different populations from south Europe was investigated. Between 1989 and 1993 we interviewed incident cases and a random population sample of controls from five centres where a cancer registry was operating, whereas we selected a sample of hospital-based cases and controls from the other three centres. We gathered information on life-long exposure to sunlight during different activities. Results are analysed for 1549 basal cell carcinoma (BCC) cases and 228 squamous cell carcinoma (SCC) cases compared with 1795 controls. We observed a statistically significant increase of risk of SCC with increasing sun exposure beyond a threshold of 70,000 cumulated hours of exposure in a lifetime. Sun exposures during work and holidays were, however, inversely correlated. Odds ratios (ORs) of SCC were up to eight or nine times the reference for the highest exposures (200,000 cumulated hours or more). BCC exhibited a 2-fold increase of risk for lower exposure (8000-10,000 cumulated hours in a lifetime) with a plateau and a slight decrease of risk for the highest exposures (100,000 cumulated hours or more). Outdoor work showed a significantly increased risk of SCC (OR 1.6 for more than 54,000 cumulated hours of exposure in a lifetime), whereas recreational activities such as sun exposure during holidays at the beach (OR 1.6 for more than 2600 cumulated hours of exposure in a lifetime) or during water sports (OR 1.6 for more than 2600 cumulated hours of exposure in a lifetime) were associated with an increased risk of BCC. Risk patterns were different in poor or good tanners with a significant risk trend for good tanners, whereas poor tanners were on a plateau of increased risk at any level of exposure. Solar radiation is associated with a risk of BCC even for relatively short periods of exposure such as during holidays and sports, whereas SCC develops later if exposure continues. The skin's ability to tan modulates the risk of BCC; subjects who tan poorly have a steady risk increase, whereas people who tan easily develop cancer only after prolonged exposures.


British Journal of Cancer (1996) 73, 1447-1454
© 1996 Stockton Press All rights reserved 0007-0920/96 $12.00

### The multicentre south European study '*Helios*' II: different sun exposure patterns in the aetiology of basal cell and squamous cell carcinomas of the skin

S Rosso[1], R Zanetti[1], C Martinez[2], M J Tormo[3], S Schraub[4], H Sancho-Garnier[5], S Franceschi[6], L Gafà[7], E Perea[2], C Navarro[3], R Laurent[8], C Schrameck[9], R Talamini[6], R Tumino[7] and J Wechsler[10]

*[1]Registro Tumori Piemonte, via S. Francesco da Paola 31, 10123 Turin, Italy; [2]Registro de Càncer de Granada, Escuela Andaluza de Salud Publica, Campus de Cartuja, 18080 Granada, Spain; [3]Registro de Càncer de Murcia, Consejeria de Sanidad y Asuntos Sociales, Ronda de Levante 11, 30008 Murcia, Spain; [4]Registre des Tumeurs du Doubs, C.H.R. Jean Minjoz, Boulevard Fleming, 25030 Besançon, France; [5]Départment de l'Information Médicale, Hôpital Gaston Doumergue, 5 Rue Hoche, 30006 Nîmes, France; [6]Centro di Riferimento Oncologico, via Pedemontana Occidentale 12, 33081 Aviano, Italy; [7]Registro Tumori di Ragusa, Piazza Igea 2, 97100 Ragusa, Italy; [8]Service de Dermatologie, Hôpital Saint Jacques, 25030 Besançon, France; [9]Service de Biostatistique, Institut Gustave Roussy, Rue Camille Desmoulins, 94805 Villejuif, France; [10]Service d'Anatomie et de Cytologie Pathologiques, Hôpital Henri Mondor, 94010 Créteil, France.*

**Summary** The role of sun exposure in development of basal cell and squamous cell carcinomas among different populations from south Europe was investigated. Between 1989 and 1993 we interviewed incident cases and a random population sample of controls from five centres where a cancer registry was operating, whereas we selected a sample of hospital-based cases and controls from the other three centres. We gathered information on life-long exposure to sunlight during different activities. Results are analysed for 1549 basal cell carcinoma (BCC) cases and 228 squamous cell carcinoma (SCC) cases compared with 1795 controls. We observed a statistically significant increase of risk of SCC with increasing sun exposure beyond a threshold of 70 000 cumulated hours of exposure in a lifetime. Sun exposures during work and holidays were, however, inversely correlated. Odds ratios (ORs) of SCC were up to eight or nine times the reference for the highest exposures (200 000 cumulated hours or more). BCC exhibited a 2-fold increase of risk for lower exposure (8000-10 000 cumulated hours in a lifetime) with a plateau and a slight decrease of risk for the highest exposures (100 000 cumulated hours or more). Outdoor work showed a significantly increased risk of SCC (OR 1.6 for more than 54 000 cumulated hours of exposure in a lifetime), whereas recreational activities such as sun exposure during holidays at the beach (OR 1.6 for more than 2600 cumulated hours of exposure in a lifetime) or during water sports (OR 1.6 for more than 2600 cumulated hours of exposure in a lifetime) were associated with an increased risk of BCC. Risk patterns were different in poor or good tanners with a significant risk trend for good tanners, whereas poor tanners were on a plateau of increased risk at any level of exposure. Solar radiation is associated with a risk of BCC even for relatively short periods of exposure such as during holidays and sports, whereas SCC develops later if exposure continues. The skin's ability to tan modulates the risk of BCC; subjects who tan poorly have a steady risk increase, whereas people who tan easily develop cancer only after prolonged exposures.

**Keywords:** basal cell carcinoma; squamous cell carcinoma; sun exposure; case-control; skin cancer

Solar radiation has been investigated as the chief risk factor for non-melanocytic skin cancer (NMSC) for many years since Molesworth (1927) suggested that sun exposure caused skin cancer (rodent ulcer). Roffo (1934) observed the effect of natural sunlight in causing tumours in rats. He reported mainly squamous cell carcinoma (SCC) occurring on hairless sites such as ear and nose. More recently a hairless mouse model has been used together with controlled emission of artificial UV radiation (Winkelman *et al.*, 1960), but the observations chiefly apply to SCC. Basal cell carcinoma (BCC) has rarely been studied in experimental animals (IARC, 1992). Rather, animal models have been based on the lack of a melanocytic protective system that can modulate the amount of radiation hitting target cells in albino mice.

In humans, the relationship between non-melanocytic skin cancer (NMSC) and solar radiation has mostly been based on descriptive studies and clinical findings. First of all, it has long been known that the anatomical site distribution of lesions greatly favours sun-exposed sites, such as head, neck and face (Levi *et al.*, 1988; Scotto *et al.*, 1983; Magnus, 1991; Marks *et al.*, 1993). With respect to ecological studies, in the northern hemisphere the highest rates are observed in northern countries owing to the phenotypic characteristics of local populations. A geographic pattern consistent with a causal role of sunlight is also observed within countries (Scotto *et al.*, 1983; Kricker *et al.*, 1994).

Measurement of the effect of sun exposure on NMSC at an individual level has been undertaken in a few studies (Gellin *et al.*, 1965; Urbach *et al.*, 1974; Hunter *et al.*, 1990; Vitasa *et al.*, 1990; Gafà *et al.*, 1991). In general, cumulative lifetime sun exposure has a clearer effect on SCC than on BCC (Vitasa *et al.*, 1990; Gafà *et al.*, 1991). The studies that considered circumstance of exposure showed that sun exposure during work was associated more strongly with SCC than BCC (Green and Battistutta, 1990; Gafà *et al.*, 1991). Exposure during non-working activities has been studied rarely in non-melanocytic skin cancer and has not generally been quantified satisfactorily, thus failing to show clear results.

Recently, a paper by Kricker *et al.* (1995*a*) put forward the hypothesis that it is intermittent sun exposure during recreational activities that has a causal role in inducing BCC. Unfortunately, the low incidence of SCC hampered similar analysis of this cancer. Older age of occurrence, anatomical site distribution of lesions and a stronger relationship with sun exposure during outdoor work suggested, however, that continuous and cumulative effects are involved in the aetiology of SCC more than intermittent sun exposure.

Correspondence: R Zanetti, Registro Tumori Piemonte, Unità di Epidemiologia dei Tumori, via San Francesco da Paola 31, 10123 Turin, Italy
Received 21 August 1995; revised 20 December 1995; accepted 8 January 1996

In 1989 we undertook a case–control study to evaluate the role of several potential risk factors on both SCC and BCC among south European populations with various skin characteristics and sun exposure habits. In this report we have examined the association of BCC and SCC with sun exposure during different activities and at different ages.

### Methods and subjects

Between November 1989 and June 1993 we recruited cases and controls in seven south European regions: Turin (north-west Italy), Trento (north-east Italy), Ragusa (Sicily), Villejuif and Créteil (Paris), Besançon (Franche-Comté, France), Murcia (south-east Spain) and Granada (Andalusia, Spain). Details on study design and interview setting are given in the accompanying paper (Zanetti *et al.*, 1996). Validation of morphological diagnoses was attained thanks to a panel of pathologists who reviewed all slides blindly.

We randomly sampled the control group from the corresponding general populations in areas covered by cancer registries. Control sampling was hospital based in Paris and Trento excluding patients with cancer or skin diseases.

### *Assessment of exposure*

Trained interviewers asked questions on sun exposure using a structured questionnaire arranged by life period (childhood, adolescence, adulthood, retirement) with separate sections on places of residence for more than 6 months, work, holidays and sports or other outdoor recreational activities. Every time subjects reported outdoor exposures, they were asked to describe these in terms of years of activity, prevalent season of exposure (warmer and cooler months), hours of exposure (amount and distribution during daylight) and usual clothing during such activities. As an aid to recall of type of clothing, questions were divided into body sections (head, trunk, upper and lower limbs, feet). We were, therefore, able to estimate amount of solar irradiation as number of hours of sun exposure received by broad body sites during different activities in a lifetime. We preferred this method of gathering information on body exposure, as asking direct questions on exposure of body site in some cases would have induced a differential recall bias. Subjects were asked to report only holidays of 1 week or more, while weekends spent outdoors were included in recreational activities. Holidays outside Europe were recorded in a separate section with more details about place, duration and amount of sun exposure during daylight.

In addition to outdoor work, we collected details on each job held for at least 6 months, providing information about type of work and industry.

### *Scales construction and data analysis*

From the available information, we derived indices of lifetime sun exposure based upon duration as well as specific indices for type of activity, period of life and part of body skin exposed. We estimated duration of exposure by summing the number of hours spent in a lifetime in a particular outdoor activity. Seasons and their different levels of sun irradiation were taken into account by assigning a weight proportional to the ratio of summer to winter irradiation in different places of exposure; on average, summer irradiation was twice the solar irradiation in winter [Data from: Bollettini Mensili di Statistica, ISTAT (Italy); Instituto Nacional de Metereologia, (Spain); Réseau Météorologique de France, (France)].

Sun exposure at specific body site was taken into account in two ways. In cancer cases we considered sun exposure specific to those body areas where the skin cancer occurred, as compared, in controls, with sun exposure in the same sites. In our study the majority of these cancers occurred on the head and the neck (80% in BCC, 75% in SCC), resulting in findings substantially similar to those for the complete set of data, and leaving few cases, especially SCC, for analysis of other sites with adequate power. As an alternative, total sun exposure can be weighted by the proportion of body surface not protected by clothing. Weights can be computed as proportions of exposed body surface to the whole body. Head and neck would represent about 9%, upper limbs 17%, lower limbs 35% and trunk 35% of the whole body surface. Weights for both the exposed body surface and season of exposure were applied in such a way that, for example, a full hour would correspond to 1 h of full exposure without clothing, whereas 6 min would correspond to 1 h exposing only the head and the face to the sun, as these body areas represent about 10% of the whole body surface.

Sun exposure indices were measured on continuous scales, but given the skewness of their distribution, we applied quartiles of distributions in exposed controls. The choice of categorising exposure indicators in this way was useful from a statistical point of view as it allowed less sparse data and more precise parameter estimation (Clayton and Hills, 1993). In the present study, the highest quartile of outdoor work entailed an elevated number of accumulated hours in a lifetime for subjects 60 years old or more who worked as farmers in south Europe, mainly in Andalusia, Murcia and Sicily. Special attention was paid to fitting regression models in the presence of outlier and leverages (Hosmer and Lemeshow, 1989).

In this analysis we evaluated the effect of sun exposure during different outdoor activities on 1549 BCC and 228 SCC cases. Odds ratios (ORs) were computed separately for BCC and SCC, referring each of these histological subtypes to the same control group made up of 1795 subjects. Confounding was first controlled adjusting for sex, age at interview and centres. Then, we adjusted each exposure for the significant pigmentary traits and skin characteristics in unconditional logistic models. In the first part of the analysis (Zanetti *et al.*, 1996), we identified a set of independent risk indicators, which included hair colour, eye colour and skin reaction to sun exposure. People who often burn rather than tan when exposed to sun showed a 2-fold risk increase for both BCC and SCC. Subjects with fair hair and blue eyes also revealed a 2-fold increase of risk for BCC, but a 4- to 8-fold increase of risk for SCC. In particular, those with red hair exhibited an OR of more than 16 for SCC. The significance of a linear trend in risk, after adjustment for confounders, was tested by including a continuous term for the variable under study in models. When the baseline of exposure corresponded to no exposure, assuming a qualitative different type of exposure, the zero level was omitted and trends in risk were assessed only in exposed subjects (Clayton and Hills, 1993). In tables we presented both tests.

### Results

Results are presented here on cumulated hours of sun exposure in a lifetime or during periods of life, since indices weighted by body exposure and seasonal solar irradiation showed similar results in all analyses by activity type.

### *Outdoor work*

Outdoor work entailed the largest number of cumulated hours of exposure. Its mean and median were about 15 times higher than those of holidays or outdoor sports. Sun exposure indices showed some variations among countries. Controls recruited by Spanish collaborating centres exhibited a higher propensity to expose themselves during outdoor work than subjects in Italy and France. Outdoor work showed no apparent effect on risk of BCC. Only one-quarter of BCC patients, as compared with 40% of SCC, fell in the highest outdoor exposure category. A similar, but higher effect in the extreme quartile was found for SCC; in addition, a significant linear trend was present in SCC cases (Table I).

*Sun exposure during holidays*

Holidays showed an opposite pattern, with an increased risk and a significant linear trend only in BCC (Table II). The paradoxical result of a protective effect for SCC, although of borderline statistical significance, could be explained by the negative correlation between outdoor work and recreational exposure. The independent effect of sun exposure indices is further explored in a later section of the results. The association between BCC and sun exposure during holidays was reinforced in an analysis restricted to holidays spent at the beach (Table III). A considerable proportion (65%) of hours of sun exposure during holidays was spent at the beach. Holidays spent in other places were not associated with significant risk increases. With respect to the period of life when holiday exposure occurred, for BCC lifetime cumulative exposure and cumulative exposure during childhood exerted similar effects (Table III), while for SCC we found an increased risk only in the lowest quartile of exposure during childhood. The lack of a similar cumulative effect for SCC might be due to the above-mentioned negative correlation with outdoor work in adulthood.

*Outdoor sports*

BCC was not associated with outdoor sports, either considering duration by itself or weighted by intensity and clothing (Table IV). On the other hand, outdoor sports seemed to protect against SCC occurrence (Table IV). Restricting analysis to sports with intense exposure, we found a significant association between BCC and water sports such as swimming, surfing, boating and sailing, although without a clear dose–response effect (Table IV). A weak and non-significant effect of sports that entail a particularly large amount of solar irradiation, such as sports practised in mountains (skiing, climbing and hiking) and in the air (flying, hang-gliding, parachuting), was present for BCC (Table IV). On the other hand, prevalence of such exposure was quite low. The negative association between outdoor sports and SCC disappeared when water sports were examined separately (Table IV).

*Dose–response relationship*

To explore further the relationship between risk of BCC or SCC and sunlight, we fitted the total number of cumulated hours of sun exposure measured on a continuous scale, including terms for centre, age, sex, hair colour, eye colour and skin reaction to sun. A log transformation of the original scale has been necessary to avoid numerical problems when treating several digit numbers and to linearise skewed continuous variables while keeping the original ratio scale of measurement. Risk of BCC and SCC were both fitted by a quadratic model but after a log transformation of the original scale; higher order terms were not statistically significant. The dose–response curve for BCC showed an initial rise of risk at relatively low doses, with a maximum at 8000–10 000 cumulated hours of exposure, followed by a plateau and then by a slight decrease (Figure 1). On the contrary, risk of SCC showed a steady and constant increase only for more than 70 000 hours of exposure. Risks were up to 8–9 times the reference level for 100 000 hours or more.

Decreasing risks of BCC at high doses can imply a complex dose–response relationship with different effects in some subgroups. Separate analysis of sun exposure in poor tanners (subjects who never tan when exposed to sun or more

**Table I** Odds ratios (ORs) of BCC and SCC by sun exposure during outdoor work in a lifetime

| *Lifetime exposure (h)* | *No. of controls* | *No. of BCCs* | *No. of SCCs* | *BCC OR[a] (95% CI)* | *SCC OR[a] (95% CI)* |
|---|---|---|---|---|---|
| <7200 | 589 | 519 | 40 | 1 (Reference) | 1 (Reference) |
| 7200–12 480 | 391 | 339 | 43 | 0.95 (0.77–1.19) | 1.04 (0.62–1.75) |
| 12 481–54 720 | 405 | 370 | 58 | 1.01 (0.81–1.25) | 1.28 (0.77–2.14) |
| 54 720+ | 410 | 321 | 87 | 0.84 (0.65–1.10) | 1.60 (0.93–2.75) |
| *P*-value (linear trend) | | | | 0.186 | 0.029 |
| *P*-value (linear trend among exposed subjects) | | | | 0.089 | 0.008 |

[a] Logistic regression estimates with terms for sex, age, centre, hair colour, eye colour and skin reaction to sun exposure.

**Table II** Odds ratios (ORs) of BCC and SCC by sun exposure during holidays in a lifetime

| *Lifetime exposure (h)* | *No. of controls* | *No. of BCCs* | *No. of SCCs* | *BCC OR[a] (95% CI)* | *SCC OR[a] (95% CI)* |
|---|---|---|---|---|---|
| Never | 580 | 425 | 100 | 1 (Reference) | 1 (Reference) |
| <280 | 310 | 281 | 39 | 1.20 (0.97–1.48) | 0.74 (0.49–1.12) |
| 280–1323 | 300 | 274 | 31 | 1.26 (1.01–1.56) | 0.65 (0.41–1.03) |
| 1324–3398 | 302 | 239 | 29 | 1.10 (0.88–1.39) | 0.61 (0.37–0.98) |
| 3398+ | 303 | 330 | 29 | 1.47 (1.18–1.83) | 0.63 (0.39–1.03) |
| *P*-value (linear trend) | | | | 0.036 | 0.047 |
| *P*-value (linear trend among exposed subjects) | | | | 0.077 | 0.660 |

[a] Logistic regression estimates with terms for sex, age, centre, hair colour, eye colour and skin reaction to sun exposure.

Table III Odds ratios (ORs) of BCC and SCC by sun exposure during holidays at the beach in a lifetime and during childhood

| Exposure (h) | No. of controls | No. of BCCs | No. of SCCs | BCC OR[a] (95% CI) | SCC OR[a] (95% CI) |
|---|---|---|---|---|---|
| Lifetime | | | | | |
| Never | 768 | 579 | 126 | 1 (Reference) | 1 (Reference) |
| <184 | 256 | 222 | 29 | 1.13 (0.91-1.40) | 0.72 (0.46-1.13) |
| 184-831 | 258 | 240 | 24 | 1.25 (1.00-1.54) | 0.59 (0.36-0.96) |
| 832-2464 | 259 | 223 | 19 | 1.19 (0.95-1.48) | 0.47 (0.27-0.80) |
| 2464+ | 254 | 285 | 30 | 1.58 (1.27-1.96) | 0.91 (0.57-1.45) |
| P-value (linear trend) | | | | <0.001 | 0.128 |
| P-value (linear trend among exposed subjects) | | | | 0.081 | 0.596 |
| In childhood | | | | | |
| Never | 1352 | 1114 | 176 | 1 (Reference) | 1 (Reference) |
| <197 | 111 | 97 | 19 | 1.05 (0.78-1.41) | 1.98 (1.14-3.44) |
| 197-714 | 111 | 113 | 10 | 1.23 (0.93-1.64) | 0.78 (0.38-1.58) |
| 715-2079 | 111 | 98 | 13 | 1.10 (0.82-1.48) | 1.15 (0.60-2.20) |
| 2079+ | 110 | 127 | 10 | 1.43 (1.09-1.89) | 0.99 (0.49-1.97) |
| P-value (linear trend) | | | | 0.005 | 0.782 |
| P-value (linear trend among exposed subjects) | | | | 0.093 | 0.194 |

[a] Logistic regression estimates with terms for sex, age, centre, hair colour, eye colour and skin reaction to sun exposure.

often burn than tan) *vs* good tanners (subjects who never or seldom burn) showed that poor tanners are at increased risk from sun exposure during beach holidays (OR = 1.4 for up to 831 h) and during water sports (OR = 2.1 for up to 2112 h) with a sort of plateau effect in the highest quartile (OR = 1.6) (Table V). Good tanners showed a linear and constant increase of risk for holidays at the beach, while water sports did not show any significant result (Table V). Risk of SCC for overall sun exposure lifelong in poor tanners exhibited a constant increase beginning at about 10 000 h of exposure, followed by a plateau at 50 000-60 000 h. In good tanners risk of SCC began to increase only above 100 000 h of exposure.

### *Independent effect of sun exposure indices*

To investigate the independent effect of sun exposure during specific outdoor activities, we tested the significance of each exposure index in a model incorporating design variables, pigmentary traits, skin characteristics and activity-specific sun exposure indices. We collapsed certain variable categories, to keep the models as parsimonious as possible and to improve their fit.

Odds ratio estimates for skin characteristics and pigmentary traits remained stable and were not influenced by the introduction of sun exposure indices. On the other hand, adjustment for host factors slightly increased the odds ratio estimates for sun exposure indices. Beach holidays and water sports emerged as independent risk factors for BCC, whereas outdoor work did the same for SCC (Table VI). Taking into consideration all sun exposure activities in the same model for SCC reduced the previous protective effect of holidays at the beach and reversed the protective effect of outdoor sports. Also, outdoor work exhibited an almost significant association with BCC after controlling for other outdoor activities. Young age at first sunburn also emerged as an independent risk factor for BCC when adjusted for sun exposure, whereas sunburns maintained their significant association with BCC only without adjustment for skin type, pigmentary traits and age at first sunburn.

### Discussion

In this study we had the opportunity to study sun exposure among a large number of BCC and SCC cases from several southern European populations with different sun exposure patterns and skin types. In a previous analysis of skin characteristics and sunburns, a different risk pattern emerged between BCC and SCC. This was also confirmed in the present analysis. Whereas SCC risk was significantly associated with sun exposure during outdoor work, recreational exposure seems more important for BCC. Outdoor work entails a more constant type of sun exposure, extended over seasons with different solar irradiation; also it allows for a regular development of natural protection through tanning and thickening of the external skin layers. In contrast, sun exposure during holidays or outdoor sports occurs during limited periods of the year, usually at weekends or during a few weeks of holiday each year; people are exposed without the opportunity to build up a natural skin protection.

Recent results from a case-control study, mostly based on prevalent cases recruited during a population survey, raised the hypothesis that intermittent sun exposure is related to BCC (Kricker *et al.*, 1995*a*). This was supported by findings on sunburns, use of sunscreens, sunbathing habits and measurements of sun exposure during recreational activities. In particular, the period of maximum risk for sun exposure was identified as before 20 years of age, although with odds ratios higher than in our study (OR = 3.8 in the highest quartile of exposure) (Kricker *et al.*, 1995*a*). With respect to the finding of increased risk early in life, the independent

**Table IV** Odds ratios (ORs) of BCC and SCC by sun exposure during outdoor sports in a lifetime

| *Exposure (h)* | *No. of controls* | *No. of BCCs* | *No. of SCCs* | *BCC OR[a] (95% CI)* | *SCC OR[a] (95% CI)* |
|---|---|---|---|---|---|
| Lifetime | | | | | |
| Never | 632 | 513 | 97 | 1 (Reference) | 1 (Reference) |
| <288 | 291 | 285 | 35 | 1.22 (0.99-1.51) | 0.43 (0.47-1.05) |
| 288-1008 | 291 | 255 | 38 | 1.10 (0.89-1.51) | 0.69 (0.45-1.06) |
| 1009-3420 | 291 | 249 | 29 | 1.07 (0.86-1.32) | 0.49 (0.31-0.78) |
| 3420+ | 290 | 247 | 29 | 1.01 (0.84-1.28) | 0.49 (0.31-0.78) |
| *P*-value (linear trend) | | | | 0.552 | 0.050 |
| *P*-value (linear trend among exposed subjects) | | | | 0.327 | 0.177 |
| During water sports | | | | | |
| Never | 1515 | 1228 | 188 | 1 (Reference) | 1 (Reference) |
| <193 | 70 | 78 | 10 | 1.39 (0.99-1.96) | 1.11 (0.55-2.25) |
| 193-770 | 70 | 88 | 11 | 1.58 (1.14-2.20) | 1.15 (0.57-2.30) |
| 771-2112 | 70 | 78 | 7 | 1.47 (1.06-2.07) | 0.58 (0.25-1.35) |
| 2112+ | 70 | 77 | 12 | 1.42 (1.01-2.00) | 1.21 (0.62-2.35) |
| *P*-value (linear trend) | | | | <0.001 | 0.567 |
| *P*-value (linear trend among exposed subjects) | | | | 0.960 | 0.094 |
| During sports in mountains and air | | | | | |
| Never | 1551 | 1307 | 207 | 1 (Reference) | 1 (Reference) |
| <140 | 61 | 65 | 5 | 1.22 (0.85-1.77) | 0.68 (0.26-1.82) |
| 140-504 | 61 | 61 | 7 | 1.14 (0.79-1.66) | 1.03 (0.44-2.40) |
| 505-1887 | 61 | 58 | 5 | 1.06 (0.72-1.54) | 0.61 (0.23-1.64) |
| 1887+ | 61 | 58 | 4 | 1.04 (0.72-1.52) | 0.50 (0.17-1.47) |
| *P*-value (linear trend) | | | | 0.438 | 0.133 |
| *P*-value (linear trend among exposed subjects) | | | | 0.542 | 0.465 |

[a] Logistic regression estimates with terms for sex, age, centre, hair colour, eye colour and skin reaction to sun exposure.

effect of young age at first sunburn in the absence of any substantial effect of number of sunburns, as found in our study, may suggest that what is relevant is not so much sunburn *per se* but rather high exposure early in life, as also shown by studies on Australian migrants (Armstrong *et al.*, 1983; Marks *et al.*, 1993). Results on the relationship between sun exposure and BCC are also very similar to those found in studies of cutaneous malignant melanoma, although ORs for outdoor work in BCC were higher than those found in melanoma (Østerlind *et al.*, 1988; Zanetti *et al.*, 1988, 1992).

Studies on SCC are fewer and seldom assess sun exposure during different activities on a quantitative basis with proper adjustment for confounding (Kricker *et al.*, 1994). Two studies found an increase of risk for working in agriculture for ten years or more (OR = 2.4, 95% CI 0.9-6.3; Gafà *et al.*, 1991) and for farmers *vs* non-farmers (OR = 1.5, 95% CI 1.2-1.8; Hogan *et al.*, 1990).

The empirical evidence of an association between 'intermittent' sun exposure and risk of BCC, as evidenced by the above-mentioned studies, was based on 'intermittence' defined as exposure during non-working days, which, in turn, was characterised by a smaller number of hours of exposure than 'constant' exposure as during outdoor work. Indeed, our results showed that the highest total irradiation for non-working activities associated with a risk increase of BCC, was below 10 000 cumulated hours of exposure, while risk increase of SCC was associated with exposure ten times higher than that of BCC. Therefore, most of the different effects of exposure patterns may be due to an additive effect of the total amount of solar radiation with a threshold specific for different epidermal cells or cells at a different growth stage. Such thresholds for cells of the basal layers may be also reached early in life, as suggested by our results on sunburns and by the increased risk for sun exposure early in life in other studies (Armstrong *et al.*, 1983; Marks *et al.*, 1989; Kricker *et al.*, 1995*a*).

Results from experiments on animals, clinical findings, descriptive epidemiological data and analytical studies suggests that, after allowance for skin characteristics, sun exposure acts on target cells, basal or squamous, with the same cumulative mechanisms, first hitting the basal layer, more prone to malignant transformation owing to its high mitotic activity. Our findings suggest that the plausible mechanism of action of sun exposure is modified by the skin's natural protection; sunlight can induce BCC if its radiation reaches the basal layers as directly as possible, even at a relatively low dose. If protection develops and sun exposure continues, subjects tend to develop SCC rather than BCC while risk of BCC reaches a plateau at high doses. Similar results were found in the Western Australia case–control study, but with a peak of risk at 35 000 hours of exposure, and then with a substantial risk decrease at high doses (Kricker *et al.*, 1995*b*). Furthermore, these findings are also in accord with a late age of occurrence of SCC and a higher proportion of BCC on the trunk which is usually less exposed to sun (Zanetti *et al.*, 1996). Natural skin protection embraces not only tanning ability, but also skin thickness.

**Figure 1** Dose–response curves (——) and 95% confidence intervals (— ——) for risk of BCC (○) and SCC (●) in relation to hours of sun exposure in a lifetime. Points fitted and displayed by tenths of the log-transformed scale in the observed range.

For example, the minimal erythema dose varies according to anatomical site, being higher in sites with a greater skin thickness (Diffey, 1982).

Furthermore, skin type also affected the shape of the dose–response relationship for BCC. In our study, as well as other studies (Kricker *et al.*, 1995*b*), poor tanners showed sensitivity at relatively low doses of solar radiation, while good tanners showed a constant increase of risk. Again, the mechanism of action reproduced what was already seen in SCC: in the case of good tanners who exposed themselves to higher doses of radiation, tanning did not provide a sufficient protection against occurrence of BCC. Otherwise, beyond a threshold of 70 000 cumulated hours of exposure, prolonged exposure to solar radiation, as during outdoor work, induces malignant transformation of cells in the squamous layer.

Measuring sun exposure over a lifetime raises several methodological issues that should be considered in interpreting these results. Direct measurement of solar irradiation starting from childhood up to cancer occurrence, which can be very late in the elderly, is not feasible on an individual basis, and general transversal measurements would be prone to ecological fallacy. On the other hand, retrospective gathering of information over several decades before interview, as in case–control studies, is a difficult exercise even for subjects with good memory. A questionnaire structured by period of life and for relevant activities, as in the present study, can help in this exercise; it was originally used in the Western Canada and Western Australia case–control studies on melanoma (Holman and Armstrong, 1984; Elwood *et al.*, 1985), but it cannot prevent differential recall bias.

The measurement issue also extends to the method of deriving indices of exposure. It is unknown whether duration of sun exposure and level of solar irradiation act on skin cancer risk with different effects and, if so, which ones. In the case of smoking, for instance, increased risk of lung cancer is much steeper with duration than with intensity of smoking habit (i.e. cigarettes per day).

**Table V** Odds ratios (ORs) of BCC in poor and good tanners by sun exposure indicators

| | *Poor tanners* | | | *Good tanners* | | |
|---|---|---|---|---|---|---|
| *Exposure (h)* | *No. of controls* | *No. of BCCs* | *OR[a] (95% CI)* | *No. of controls* | *No. of BCCs* | *OR[a] (95% CI)* |
| During beach holidays | | | | | | |
| Never | 460 | 386 | 1 (Reference) | 302 | 187 | 1 (Reference) |
| <184 | 140 | 153 | 1.26 (0.96–1.66) | 115 | 69 | 0.98 (0.69–1.39) |
| 184–831 | 127 | 148 | 1.36 (1.03–1.81) | 131 | 91 | 1.11 (0.79–1.56) |
| 832–2464 | 135 | 126 | 1.05 (0.78–1.43) | 124 | 97 | 1.26 (0.89–1.77) |
| 2464+ | 122 | 151 | 1.40 (1.04–1.89) | 132 | 132 | 1.54 (1.12–2.12) |
| *P*-value (linear trend) | | | 0.039 | | | 0.003 |
| *P*-value (linear trend among exposed subjects) | | | 0.764 | | | 0.008 |
| During water sports | | | | | | |
| Never | 843 | 747 | 1 (Reference) | 666 | 472 | 1 (Reference) |
| <193 | 34 | 46 | 1.39 (0.88–2.21) | 36 | 32 | 1.37 (0.83–2.26) |
| 193–770 | 35 | 60 | 1.92 (1.24–2.96) | 35 | 28 | 1.20 (0.72–2.02) |
| 771–2112 | 33 | 57 | 2.09 (1.33–3.27) | 37 | 21 | 0.91 (0.52–1.59) |
| 2112+ | 39 | 54 | 1.61 (1.04–2.49) | 31 | 23 | 1.17 (0.67–2.05) |
| *P*-value (linear trend) | | | <0.001 | | | 0.471 |
| *P*-value (linear trend among exposed subjects) | | | 0.660 | | | 0.503 |

[a] Logistic regression estimates with terms for sex, age, centre, hair colour, eye colour.

**Table VI** Odds ratios (ORs) of BCC and SCC including pigmentary traits, skin characteristics, outdoor activities and adjusting for age, sex and centre

| | *No. of controls* | *No. of BCCs* | *No. of SCCs* | *BCC OR (95% CI)* | *SCC OR (95% CI)* |
|---|---|---|---|---|---|
| Hair colour | | | | | |
| Black | 154 | 99 | 12 | 1 (Reference) | 1 (Reference) |
| Brown | 699 | 544 | 80 | 1.16 (0.87–1.54) | 1.56 (0.79–2.86) |
| Light brown | 597 | 514 | 67 | 1.21 (0.90–1.62) | 1.63 (0.84–3.17) |
| Blonde | 253 | 257 | 30 | 1.24 (0.89–1.71) | 1.73 (0.83–3.61) |
| Light blonde | 81 | 121 | 29 | 1.70 (1.14–2.54) | 5.49 (2.49–12.07) |
| Red | 11 | 16 | 10 | 1.34 (0.57–3.12) | 14.44 (4.72–44.18) |
| Eye colour | | | | | |
| Black/dark brown | 542 | 332 | 39 | 1 (Reference) | 1 (Reference) |
| Blue/hazel/grey/green | 1253 | 1217 | 189 | 1.39 (1.17–1.65) | 1.55 (1.05–2.30) |
| Skin reaction to sun exposure | | | | | |
| Tan, no burn | 518 | 299 | 44 | 1 (Reference) | 1 (Reference) |
| Burn then tan | 1088 | 943 | 71 | 1.48 (1.24–1.76) | 1.42 (0.97–2.07) |
| Burn, never tan | 184 | 299 | 43 | 2.81 (2.18–3.62) | 2.02 (1.20–3.40) |
| Age at first sunburn | | | | | |
| More than 15 years old or never | 1741 | 1458 | 218 | 1 (Reference) | 1 (Reference) |
| 15 years old or less | 54 | 91 | 10 | 1.45 (1.00–2.12) | 1.35 (0.62–2.97) |
| Number of sunburns in a lifetime | | | | | |
| Fewer than three | 1715 | 1429 | 217 | 1 (Reference) | 1 (Reference) |
| Three or more | 80 | 102 | 11 | 1.05 (0.86–1.42) | 0.94 (0.55–1.62) |
| Outdoor work in a lifetime (h) | | | | | |
| <7200 | 589 | 519 | 40 | 1 (Reference) | 1 (Reference) |
| 7200–54 720 | 796 | 709 | 101 | 1.02 (0.84–1.24) | 1 (Reference) |
| 54 720+ | 410 | 321 | 87 | 1.00 (0.78–1.30) | 1.6 (1.04–2.47) |
| Holidays at beach in a lifetime (h) | | | | | |
| Never | 768 | 579 | 126 | 1 (Reference) | 1 (Reference) |
| <2464 | 773 | 685 | 72 | 1.12 (0.95–1.32) | 1 (Reference) |
| 2464+ | 254 | 285 | 30 | 1.47 (1.18–1.84) | 0.92 (0.82–1.04) |
| Water sports in a lifetime (h) | | | | | |
| Never | 1515 | 1228 | 188 | 1 (Reference) | 1 (Reference) |
| <2112 | 210 | 244 | 28 | 1.45 (1.18–1.79) | 1.03 (0.65–1.62) |
| 2112+ | 70 | 77 | 12 | 1.47 (1.04–2.07) | 1.43 (0.73–2.79) |

Considering that recall bias could be relevant, in this first analysis we chose to relate exposure not to the specific anatomical site of the lesion as in other studies (Kricker *et al.*, 1995*a*, *b*). It must, however, be borne in mind that the most frequent anatomical site was head, neck and face in about 77% of BCC and 70% of SCC, leaving few cases in other sites for analysis (Zanetti *et al.*, 1996).

In addition, testing for the combined effect of specific sun exposures led us to deal with collinearity of some indices or with small sample size in some covariate patterns. Small numbers of subjects with very high values can bias regression coefficients. On the other hand, the extreme points of distribution are often the most interesting situation to explore as in the case of people working outdoors such as farmers, fishermen or bricklayers.

In summary, sun exposure can induce BCC at relatively low doses, whereas SCC develops only if people expose themselves to higher doses for a prolonged time. Skin type modulates the response; people who tan poorly will have an increased risk even if moderately exposed, whereas prolonged sun exposure can cause BCC even in good tanners or, at even higher levels of exposure, SCC.

**Acknowledgements**
This study has been supported by a research grant from Europe Against Cancer (contract nos. 890139, 910539, 920584) and by Associazione Italiana per la Ricerca sul Cancro (AIRC), Italy; Fondo de Investigacion Sanitaria de la Securidad Social (FISS), Spain (contract no. 90E0720); Ligue Nationale Contre le Cancer, France; Consiglio Nazionale delle Ricerche (CNR), Italy; Institut National de la Santé et de la Recerche Médicale (INSERM), France. We thank Mrs M Casale for her valuable help in preparing the questionnaire and in training interviewers and Dr BK Armstrong for his useful comments on the manuscripts. We are also grateful to the numerous colleagues, dermatologists, pathologists and general practitioners who allowed access to patients and histological material.

### References

ARMSTRONG BK, WOODINGS T, STENHOUSE NS AND MCCALL MG. (1983). *Mortality from Cancer in Migrants to Australia 1962–1971*. University of Western Australia: Perth.

CLAYTON D AND HILLS M. (1993). *Statistical models in Epidemiology*, pp. 254–258. Oxford University Press: Oxford.

DIFFEY BL. (1982). The consistency of studies of ultraviolet erythema in normal human skin. *Phys. Med. Biol.*, **27**, 715–720.

ELWOOD JM, GALLAGHER RP, HILL GB AND PEARSON JCG. (1985). Cutaneous melanoma in relation to intermittent and constant sun exposure: the Western Canada Melanoma Study. *Int. J. Cancer*, **35**, 427–433.

GAFÀ L, FILIPAZZO MG, TUMINO R, DARDANONI G, LANZARONE F AND DARDANONI L. (1991). Risk factors of nonmelanoma skin cancer in Ragusa, Sicily: a case–control study. *Cancer Causes Control*, **2**, 395–399.

GELLIN GA, KOPF AW AND GARFINKEL L. (1965). Basal cell epithelioma. A controlled study of associated factors. *Arch. Dermatol.*, **91**, 38–45.

GREEN AC AND BATTISTUTTA D. (1990). Incidence and determinants of skin cancer in a high-risk Australian population. *Int. J. Cancer*, **46**, 356–361.

HOGAN DJ, LANE PR AND GRAN L. (1990). Risk factors for squamous cell carcinoma of the skin in Saskatchewan, Canada. *J. Dermatol. Sci.*, **1**, 97–100.

HOLMAN CDJ AND ARMSTRONG BK. (1984). Cutaneous malignant melanoma and indicators of total accumulated exposure to the sun: an analysis separating histogenetic types. *J. Natl Cancer Inst.*, **73**, 75–82.

HOSMER DW AND LEMESHOW S. (1989). *Applied Logistic Regression*. Wiley series in probability and mathematical statistics. John Wiley & Sons: New York.

HUNTER DJ, COLDITZ GA, STAMPFER MJ, ROSNER B, WILLET WC AND SPEIZER FE. (1990). Risk factors for basal cell carcinoma in a prospective cohort of women. *Ann. Epidemiol.*, **1**, 13–23.

INTERNATIONAL AGENCY FOR RESEARCH ON CANCER. (1992). *IARC Monographs on the Evaluation of Carcinogenic Risks to Humans, Vol. 55, Solar and Ultraviolet Radiation*. IARC: Lyon.

KRICKER A, ARMSTRONG BK AND ENGLISH DR. (1994). Sun exposure and non-melanocytic skin cancer. *Cancer Causes Control*, **5**, 357–392.

KRICKER A, ARMSTRONG BK, ENGLISH DR AND HEENAN PJ. (1995*a*). Does intermittent sun exposure cause basal cell carcinoma? A case–control study in Western Australia. *Int. J. Cancer*, **60**, 489–494.

KRICKER A, ARMSTRONG BK, ENGLISH DR AND HEENAN PJ. (1995*b*). A dose–response curve for sun exposure and basal cell carcinoma. *Int. J. Cancer*, **60**, 482–488.

LEVI F, FRANCESCHI S, TE V, RANDIMBISON L AND LA VECCHIA C. (1988). Descriptive epidemiology of skin cancer in the Swiss canton of Vaud. *Int. J. Cancer*, **42**, 811–816.

MAGNUS K. (1991). The Nordic profile of skin cancer incidence. A comparative epidemiological study of the three main types of skin cancer. *Int. J. Cancer*, **47**, 12–19.

MARKS R, JOLLEY D, DOREVITCH AP AND SELWOOD TS. (1989). The incidence of non-melanocytic skin cancers in an Australian population: results of a five-year prospective study. *Med. J. Aust.*, **150**, 475–478.

MARKS R, STAPLES M AND GILES GG. (1993). Trends in non-melanocytic skin cancer treated in Australia: the second national survey. *Int. J. Cancer*, **53**, 585–590.

MOLESWORTH EH. (1927). Rodent Ulcer. *Med. J. Aust.*, **1**, 878–895.

ØSTERLIND A, TUCKER MA, STONE BJ AND JENSEN OM. (1988). The Danish case–control study of cutaneous malignant melanoma. II. Importance of UV-light exposure. *Int. J. Cancer*, **42**, 319–324.

ROFFO AH. (1934). Cancer and the sun: carcinomas and sarcomas caused by the action of the sun *in toto* ( in French) . *Bull. Assoc. Fr. Étude Cancer*, **23**, 590–616.

SCOTTO J, FEARS TR AND FRAUMENI JF. (1983). *Incidence of Nonmelanoma Skin Cancer in the United States*. NIH Publ. No. 83–2433. National Cancer Institute: Bethesda.

URBACH F, EPSTEIN JH AND FORBES PD. (1974). Ultraviolet carcinogenesis: experimental, global and genetic aspects. In *Sunlight and Man. Normal and Abnormal Photobiologic Responses*, Pathak MA, Harber LC, Seiji M and Kukita A. (eds) pp. 259–283. University of Tokyo Press: Tokyo.

VITASA BC, TAYLOR HR, STRICKLAND PT, ROSENTHAL FS, WEST S, ABBEY H, NG SK, MUNOZ B AND EMMET EA. (1990). Association of nonmelanoma skin cancer and actinic keratosis with cumulative solar ultraviolet exposure in Maryland watermen. *Cancer*, **65**, 2811–2817.

WINKELMAN RK, BALDES EJ AND ZOLLMAN PE. (1960). Squamous cell tumours induced in hairless mice with ultraviolet light. *J. Invest. Dermatol.*, **34**, 131–138.

ZANETTI R, ROSSO S, FAGGIANO F, ROFFINO R, COLONNA S AND MARTINA G. (1988). A case–control study on cutaneous malignant melanoma in the province of Torino, Italy (in French). *Rev. Épidém. et Santé Publ.*, **36**, 309–317.

ZANETTI R, FRANCESCHI S, ROSSO S, COLONNA S AND BIDOLI E. (1992). Cutaneous melanoma and sunburns in childhood in a southern European population. *Eur. J. Cancer*, **28A**, 1172–1176.

ZANETTI R, ROSSO S, MARTINEZ C, NAVARRO C, SCHRAUB S, SANCHO-GARNIER H, FRANCESCHI S, GAFÀ L, PEREA E, TORMO MJ, LAURENT R, SCHRAMECK C, CRISTOFOLINI M, TUMINO R AND WECHSLER J. (1996). The multicentre South European study 'HELIOS' I. Skin characteristics and sunburns in basal cell and squamous cell carcinomas of the skin. *Br. J. Cancer*, **73**, 1440–1446.